# Mild Botulism From Illicitly Brewed Alcohol in a Large Prison Outbreak in Mississippi

**DOI:** 10.3389/fpubh.2021.716615

**Published:** 2021-08-24

**Authors:** Mariel Marlow, Leslie Edwards, Lindsey McCrickard, Louise K. Francois Watkins, Jannifer Anderson, Sheryl Hand, Kathryn Taylor, Janet Dykes, Paul Byers, Kevin Chatham-Stephens

**Affiliations:** ^1^Centers for Disease Control and Prevention, Atlanta, GA, United States; ^2^Mississippi State Department of Health, Jackson, MS, United States

**Keywords:** botulism, alcohol, prison, outbreak, mild illness, cranial nerve palsies, pruno

## Abstract

Botulism is typically described as a rapidly progressing, severe neuroparalytic disease. Foodborne botulism is transmitted through consuming food or drink that has been contaminated with botulinum toxin. During a botulism outbreak linked to illicitly brewed alcohol (also known as “hooch” or “pruno”) in a prison, 11 (35%) of 31 inmates that consumed contaminated hooch had mild illnesses. This includes 2 inmates with laboratory confirmed botulism. The most frequently reported signs and symptoms among the 11 patients with mild illness included dry mouth (91%), hoarse voice (91%), difficulty swallowing (82%), fatigue (82%), and abdominal pain (82%). Foodborne botulism is likely underdiagnosed and underreported in patients with mild illness. Botulism should be considered on the differential diagnosis for patients with cranial nerve palsies.

## Introduction

Foodborne botulism is a potentially fatal neuroparalytic illness that occurs through ingestion of food or drinks contaminated with a neurotoxin produced most frequently by *Clostridium botulinum* (1). The seven botulinum toxins (A–G) inhibit acetylcholine release at the neuromuscular junction, resulting in cranial nerve palsies that may progress to descending paralysis including respiratory failure (2). Botulinum antitoxin (BAT) is indicated for suspected botulism patients with progressive illness and prevents disease progression by binding to circulating toxin (3).

Most botulism cases are identified when patients come to a hospital with severe illness including descending paralysis. For example, 66% (204/332) of non-infant botulism patients in the United States reported to CDC during 2002–2015 had respiratory failure requiring mechanical ventilation (4). Less severe cases not requiring hospitalization have been reported in outbreaks; however, published reports frequently focus on more severely affected cases, resulting in a paucity of information about less severe cases (5–8).

Botulism outbreaks in prisons linked to the consumption of home-brewed alcohol, also known as pruno or hooch, have previously been reported in California, Utah, and Arizona (9–11). In each of these outbreaks, pruno was prepared using a combination of potatoes along with other available ingredients including fruit, jelly, ketchup, bread, and/or sugar. This report describes mild botulism cases identified during an investigation at a prison in Mississippi in 2016 (12). Describing mild botulism is important because (1) identification of any botulism patient may herald an outbreak; (2) improved reporting of botulism cases can enhance surveillance and prevention efforts; and (3) raising awareness among health professionals of the clinical spectrum could improve clinical diagnosis and avoid time and costs associated with evaluation and treatment for other conditions.

## Methods

On June 9, 2016, the Mississippi State Department of Health (MSDH) was notified about a possible outbreak of botulism among inmates at a federal prison in Mississippi. State and local public health authorities interviewed inmates about food and beverages consumed since June 1, 2016. MSDH and CDC worked with the Bureau of Prisons (BoP) to identify inmates that were at risk for botulism within the prison. The BoP granted permission to interview inmates that had clinical signs of botulism and/or admitting drinking prison-brewed alcohol. Prison staff worked to identify and recruit these inmates and CDC and MSDH completed the interviews. To maintain inmates' privacy, the inmates were assigned unique ID numbers, and data collected did not include inmates' names or federal register numbers. Ethical review and approval was not required for the study on human participants, per the local legislation and institutional requirements.

Serum samples were tested by EndoPEP mass spectroscopy (MS) and stool samples were tested by mouse bioassay for botulinum toxin (13). Further details about the epidemiologic investigation have previously been published (12). For this analysis, we defined severe botulism as progressive neurological signs and symptoms that warranted treatment with botulism antitoxin (BAT) and mild botulism as non-progressing signs and symptoms that did not warrant treatment with BAT (14). We abstracted data from questionnaires, clinic notes, and hospital charts.

## Results

During June 6–8, 2016, five patients presented to a Mississippi prison clinic with cranial nerve palsies, weakness, and shortness of breath. By the end of the outbreak, 31 patients with suspected, probable, or laboratory-confirmed botulism were identified, and all 31 patients reported exposure to hooch.

Among the 31 patients, 20 (65%) had severe botulism and 11 (35%) had mild botulism. All 20 patients with severe botulism were hospitalized, and nine (45%) required intubation. All 11 mild botulism patients were men; their median age was 37 years (range: 23–47). One (9%) patient was hospitalized, and one (9%) was evaluated in an emergency department. A variety of signs and symptoms were reported among the mild botulism patients, including abdominal pain and diarrhea, cranial nerve palsies, shortness of breath, and subjective weakness (Table 1). The frequency of most symptoms did not differ significantly between patients with mild botulism and patients with severe botulism. For example, most (*n* = 6; 55%) mild botulism patients reported shortness of breath and most (*n* = 14; 74%) patients with severe botulism reported shortness of breath. Mild botulism patients were asked about their general frequency of consuming pruno (e.g., daily, weekly, monthly, less than monthly, or never) and were specifically asked about drinking pruno each day from June 1 until June 17 or the date symptoms began. Two patients refused to answer the question as to the general frequency of consuming pruno and the response among the remaining nine patients included daily (1, 11%), weekly (2, 22%), and less than monthly (6, 67%). When asked about specific dates they drank pruno from June 1 to the date of onset of symptoms, eight of 11 patients reported drinking pruno just once in that period, two patients reported drinking pruno twice and one patient reported drinking pruno on 6 of the 7 days before the onset of symptoms.

**Table 1:** Signs and symptoms reported by patients with mild botulism (*N* = 11) and patients with severe botulism (*N* = 19)^*^ in a large prison outbreak linked to illicitly brewed alcohol—Mississippi, 2016^†^.

**Symptom**	**Patients with mild botulism *n* (%)**	**Patients with severe botulism *n* (%)**
Dry mouth	10 (91)	17 (89)
Hoarse voice	10 (91)	16 (84)
Difficulty swallowing	9 (82)	19 (100)
Fatigue	9 (82)	17 (89)
Abdominal pain	9 (82)	8 (42)
Blurred vision^‡^	8 (73)	19 (100)
Dizziness	8 (73)	18 (95)
Diarrhea	8 (73)	9 (47)
Weakness^‡^	7 (64)	19 (100)
Slurred speech	6 (55)	16 (84)
Nausea	6 (55)	15 (79)
Shortness of breath	6 (55)	14 (74)

* *Questionnaire was not obtained for one patient with severe disease*.

† *Ordered by frequency reported by patients with mild botulism*.

‡ *Statistically significant difference, P < 0.05, Fisher's Exact Test*.

Seven (64%) of the mild botulism patients submitted serum and botulinum toxin type A was identified in sera of two patients tested by EndoPEP-MS. A stool sample was tested by mouse bioassay for one mild botulism patient and botulinum toxin was not identified. The two mild botulism patients with positive laboratory tests for botulism were evaluated in a hospital after the initial five patients with severe botulism were identified. The first patient, who was receiving levothyroxine for hypothyroidism, was evaluated at the emergency department on June 9 complaining of blurred vision and dysphagia (difficulty swallowing). He was not admitted because he did not have progressive signs or symptoms, extremity weakness, or respiratory distress. The second patient was admitted to the hospital on June 11 with blurred vision, dizziness, dysphagia, shortness of breath, and subjective weakness. He was discharged 48 h later without any worsening of his illness. When interviewed on June 16, both patients reported or were observed to have mild, non-progressive botulism signs and symptoms (e.g., blurred vision, hoarseness, slurred speech).

Mild botulism patients had a greater median number of days from hooch exposure to presentation at the clinic (7 days, range: 4–10) than patients with severe botulism (4 days; range: 2–5) (*P* = 0.001). Patients with severe botulism reported more hooch consumption (3.1 cups) than mild botulism patients (1.5 cups) (*P* = 0.02).

## Discussion

These 11 patients with mild botulism represented more than one-third of all patients in the outbreak, which was the largest U.S. botulism outbreak since 1978 (15). Among published outbreaks from 1920 to 2014, 15% of botulism toxin type A cases were not hospitalized (16). The greater proportion of non-hospitalized patients in this outbreak supports the likelihood of underreporting of mild illness, especially in non-institutionalized populations where case ascertainment may be more difficult. These findings, especially the documentation of laboratory-confirmed botulism among patients with mild illness, highlight the broad clinical spectrum of botulism. Patients with mild signs and symptoms of botulism might not seek care or might not be diagnosed with botulism if they do seek care, resulting in botulism cases not being identified. Due to the limited nature and lack of progression of signs and symptoms reported by mild botulism patients, none were treated with BAT. The decision regarding BAT treatment is typically made by the patient's clinical team following a consultation with their state health department and CDC. BAT is currently FDA approved only for treatment in symptomatic patients but could be given to asymptomatic patients with a known exposure as part of a CDC research protocol.

Prior botulism outbreak investigations have documented cases of mild illness, including cases reporting gastrointestinal features (e.g., nausea, vomiting) with or without cranial nerve signs or symptoms (7, 8). Multiple factors may influence the severity of botulism, including toxin type and dose, host susceptibility, mode of transmission, and immunity from repeated exposure (8). The subjective nature of symptoms may raise concern for malingering in an inmate population. For example, during the investigation, interviewers raised the concern that inmates may have been emphasizing they had visual symptoms in interviews after observing other inmates returning from the hospital wearing sunglasses (due to pupillary dilation and light sensitivity). However, the presence of botulinum toxin in the sera of two mild botulism patients indicates malingering was not responsible for all mild cases. A possible source of bias for this information is that inmates may not have been completely forthcoming about their history of consuming pruno as this is an unapproved activity in the prison. Recall bias may have also been present as inmates may have had difficulty remembering which days that they drank pruno.

Time from exposure to specimen collection, fewer specimens tested, and lower toxin dose could have resulted in fewer mild botulism cases having positive laboratory results. In addition, low levels of botulinum toxin in human serum may be enough to cause symptomatic disease but may be below the mouse bioassay limit of detection. So it is not uncommon for patients fitting the description of botulism to test negative (16, 17).

## Conclusions

Clinicians should consider botulism when evaluating a patient with symmetrical cranial nerve palsies and should assess the patient for descending weakness and respiratory involvement. If another diagnosis does not seem likely, clinicians should immediately contact CDC through their local or state health department for a clinical consultation to help determine the likelihood of botulism and the need for urgent release of BAT by CDC. The decision to administer BAT is based on clinical presentation because paralysis can progress rapidly, BAT is more effective the earlier it is given, and laboratory confirmation can take days to weeks. Knowledge of the clinical spectrum of botulism may help clinicians, prison staff, and public health officials investigating an outbreak identify and monitor patients for disease progression. Some botulism patients may have mild illness without progression and may not require BAT; however, the clinical features that predict which patients will progress and should be treated with BAT are unknown. Suspicion of botulism may reduce the need for diagnostic tests and therapeutic procedures for other conditions that may mimic botulism (e.g., Guillain-Barré syndrome, myasthenia gravis). Confirming the diagnosis of botulism may also aid in understanding a patient's prognosis.

Timely identification of botulism cases, including mild botulism, has many potential benefits, including helping improve patient care, alerting clinicians, and public health officials to a previously unrecognized outbreak, and providing additional epidemiologic information to help stop a recognized outbreak.

## Data Availability Statement

The raw data supporting the conclusions of this article will be made available by the authors, with protections in place for personally identifiable information (PII).

## Ethics Statement

The project was reviewed by CDC's Human Subjects Protection Committee and determined that it did not meet the definition of research under 45 CFR 46.102d. Informed consent was obtained from all study participants.

## Author Contributions

All authors listed have made a substantial, direct and intellectual contribution to the work, and approved it for publication.

## Conflict of Interest

The authors declare that the research was conducted in the absence of any commercial or financial relationships that could be construed as a potential conflict of interest.

## Publisher's Note

All claims expressed in this article are solely those of the authors and do not necessarily represent those of their affiliated organizations, or those of the publisher, the editors and the reviewers. Any product that may be evaluated in this article, or claim that may be made by its manufacturer, is not guaranteed or endorsed by the publisher.
